# Supplementary material for: Toward Modeling the Structure of Electrolytes at Charged Mineral Interfaces Using Classical Density Functional Theory
Source: J Phys Chem B. 2024 Apr 16;128(16):3981–96. doi: 10.1021/acs.jpcb.3c08045 (PMC11056995; doi:10.1021/acs.jpcb.3c08045)
Supplement: Supplementary file 1 — jp3c08045_si_001.pdf [file jp3c08045_si_001.pdf]

Supporting Information: Toward Modeling the Structure of Electrolytes at Charged Mineral Interfaces using Classical Density Functional Theory

Thomas Petersen[†]

[†]*Sonny Astani Department of Civil and Environmental Engineering*

University of Southern California, Los Angeles, California 90089 USA

E-mail: thomasp3@usc.edu

Phone: +1 (928) 210-2088

Model Development

Calibrated coefficients for effective packing density used with Sutherland potential

The following equation provides the coefficients, c_1 and c_2, to calculate the effective packing fraction $\bar{\eta}_{\text{eff}}$ in Eq.(19) of the main text:

$$\begin{bmatrix} c_1 \\ c_2 \end{bmatrix} = \begin{bmatrix} -0.943973 & 0.422543 & -0.0371763 & 0.00116901 \\ 0.370942 & -0.173333 & 0.0175599 & -0.000572729 \end{bmatrix} \begin{bmatrix} 1 \\ \lambda_i \\ \lambda_i^2 \\ \lambda_i^3 \end{bmatrix}. \tag{S1}$$

The coefficients were originally presented by Gil-Villegas et al. in Ref.(S1).

iSAFT model for the free energy of short-range association interactions

The iSAFT model differentiates itself from the model by Yu and Wu by abstaining from direct use of the FMT densities to evaluate the free energy and association within the mass-action laws. Instead, the iSAFT model expresses the Helmholtz free energy for particle association as

$$\beta \mathcal{F}_{\text{assoc}}^{\text{iSAFT}} = \int \mathrm{d}\boldsymbol{r} \left\{ \sum_i \rho_i(\boldsymbol{r}) \sum_\alpha \left[\ln(\chi_\alpha^{(i)}) + \frac{1-\chi_\alpha^{(i)}}{2} \right] \right\}, \tag{S2}$$

and bonding between sites is calculated from

$$\chi_{e_0}^{\text{w}} \left(1 + \int \mathrm{d}\boldsymbol{r'} \left[2\rho_{\text{w}} \chi_{\text{H}}^{\text{w}} \Delta_{e_0 \cdot \text{H}} + M_+ \rho_+ \chi^+ \Delta_{e_0 \cdot +} \right] \right) = 1 \tag{S3a}$$

$$\chi_{\text{H}}^{\text{w}} \left(1 + \int \mathrm{d}\boldsymbol{r'} \left[2\rho_{\text{w}} \chi_{e_0}^{\text{w}} \Delta_{e_0 \cdot \text{H}} + M_- \rho_- \chi^- \Delta_{\text{H} \cdot -} \right] \right) = 1 \tag{S3b}$$

$$\chi^+ \left(1 + \int \mathrm{d}\boldsymbol{r'} \left[M_+ \rho_{\text{w}} \chi_{e_0}^{\text{w}} \Delta_{e_0 \cdot +} \right] \right) = 1 \tag{S3c}$$

$$\chi^- \left(1 + \int \mathrm{d}\boldsymbol{r'} \left[M_- \rho_{\text{w}} \chi_{\text{H}}^{\text{w}} \Delta_{\text{H} \cdot -} \right] \right) = 1. \tag{S3d}$$

As is seen from the equations above, association is evaluated by integrating the density of eligible sites within reach of sites on other particles and statistically averaging their likelihood to bond. Because $\chi_\alpha^{(i)}$ is included in the integrand when averaging the site-to-site contacts, it is expected that the iSAFT procedure preserves more of the spatial correlation between site bonding and particle densities than the Yu-Wu model. Yet, as will be seen, because sites are not fixed on the particles' surfaces – instead being *smeared* over them – the iSAFT procedure leads to an accumulation of bonding near particle contacts and an overprediction in the amplitude of the density peaks; similar observations have been made in comparative

studies in Refs.[S2,S3]

As before, the interaction parameter is measured by integrating and approximating the two-point direct correlation function to find

$$\Delta^{\text{iSAFT}}_{\alpha\cdot\beta}(\boldsymbol{r},\boldsymbol{r}') = \bar{y}^{(i,j)}_{\text{hs}}(\boldsymbol{r},\boldsymbol{r}')\sin^4(\theta_{\text{c}}/2)\left[\exp\left(\beta\epsilon_{\alpha\cdot\beta}\right)-1\right]. \tag{S4}$$

Here, the hard-sphere contact correlation value is calculated, as was done in Yu-Wu, using the Carnahan-Starling equation for mixtures,[S4–S7] though it takes a slightly modified form:

$$y^{(i,j)}_{\text{hs}}(\{\varsigma_\alpha(\boldsymbol{r})\}) = \frac{1}{1-\varsigma_3} + \frac{\sigma_i\sigma_j}{2(\sigma_i+\sigma_j)}\frac{\varsigma_2}{(1-\varsigma_3)^2} + \frac{1}{18}\left(\frac{\sigma_i\sigma_j}{\sigma_i+\sigma_j}\right)^2\frac{\varsigma_2^2}{(1-\varsigma_3)^3}. \tag{S5}$$

In the equation above, the density variables $\varsigma_3 = \sum_i \bar{\rho}_i(\pi\sigma_i^3/6)$ and $\varsigma_2 = \sum_i \bar{\rho}_i(\pi\sigma_i^2)$ are calculated from

$$\bar{\rho}_i(\boldsymbol{r}) = \left(\frac{4\pi\sigma_i^3}{3}\right)^{-1}\int_{|\boldsymbol{r}-\boldsymbol{r}'|<\sigma_i} \mathrm{d}\boldsymbol{r}'\left[\Theta(|\boldsymbol{r}-\boldsymbol{r}'|-\sigma_i)\rho_i(\boldsymbol{r}')\right], \tag{S6}$$

where the convolution is carried out over a spherical volume demarcated by the particles distance of closest approach between particle centers. In estimating the interaction parameter between two location, the geometric average of Eq.(S5) is chosen

$$\bar{y}^{(i,j)}_{\text{hs}}(\boldsymbol{r},\boldsymbol{r}') = \sqrt{y^{(i,j)}_{\text{hs}}(\{\varsigma_\alpha(\boldsymbol{r})\})\times y^{(i,j)}_{\text{hs}}(\{\varsigma_\alpha(\boldsymbol{r}')\})}, \tag{S7}$$

as a measure of the reference two-point correlation.

As an example of how site-association is evaluated, Eq.(S3c) – the mass-action law for the cation sites – is simplified as follows for a planar geometry:

$$\chi^+(x)\left(1+M_+K_{ij}\left[\exp\left(\beta\epsilon_{\alpha\cdot\beta}\right)-1\right]\frac{1}{2\sigma_{\text{w}+}}\int_{x-\sigma_{\text{w}+}}^{x+\sigma_{\text{w}+}}\mathrm{d}x'\left[\bar{y}^{(i,j)}_{\text{hs}}(x,x')\rho_{\text{w}}(x')\chi^{\text{w}}_{e_0}(x')\right]\right) = 1, \tag{S8}$$

where the $(1/2\sigma_{\text{w}+})$ prefactor is used to normalize the integral and ensures dimensional

consistency, and $\sigma_{\mathrm{w+}}$ is the distance of closest approach between a water molecule and cation.

Simplified expression for the excess chemical potential due to particle association

Michelsen and Hendriks showed that the variational derivative of Wertheim's excess free energy due to particle association – required in evaluating $c_i^{(1)}(\boldsymbol{r})$ in Eq.(5) of the main text – can be mathematically simplified.[S8] Adopting their expression for the Yu-Wu and iSAFT models produces the following two equations:

$$\begin{aligned}\frac{\delta\beta\mathcal{F}_{\mathrm{assoc}}^{\mathrm{Y-W}}}{\delta\rho_i(\boldsymbol{r})} &= \sum_{\alpha\in\Gamma_i}\frac{\delta(n_{0,i}\zeta_i)}{\delta\rho_i(\boldsymbol{r})}\left[\ln(\chi_\alpha(\boldsymbol{r}))\right] - \frac{1}{2}\Bigg[\sum_i\sum_j(n_{0,i}\zeta_i)(n_{0,j}\zeta_j) \\ &\quad\times\sum_{\alpha\in\Gamma_i}\sum_{\beta\in\Gamma_j}\chi_\alpha^{(i)}(\boldsymbol{r}')\chi_\beta^{(j)}(\boldsymbol{r}'')\left(\frac{\delta\Delta_{\alpha\cdot\beta}(\boldsymbol{r}',\boldsymbol{r}'')}{\delta\rho_i(\boldsymbol{r})}\right)\Bigg]\end{aligned} \tag{S9}$$

and

$$\begin{aligned}\frac{\delta\beta\mathcal{F}_{\mathrm{assoc}}^{\mathrm{iSAFT}}}{\delta\rho_i(\boldsymbol{r})} &= \sum_{\alpha\in\Gamma_i}\left[\ln(\chi_\alpha(\boldsymbol{r}))\right] - \frac{1}{2}\int\int \mathrm{d}\boldsymbol{r}'\mathrm{d}\boldsymbol{r}''\Bigg[\sum_j\sum_k\rho_i(\boldsymbol{r}')\rho_j(\boldsymbol{r}'') \\ &\quad\times\sum_{\alpha\in\Gamma_i}\sum_{\beta\in\Gamma_j}\chi_\alpha^{(i)}(\boldsymbol{r}')\chi_\beta^{(j)}(\boldsymbol{r}'')\left(\frac{\delta\Delta_{\alpha\cdot\beta}(\boldsymbol{r}',\boldsymbol{r}'')}{\delta\rho_i(\boldsymbol{r})}\right)\Bigg].\end{aligned} \tag{S10}$$

These expressions were used to evaluate the association contributions to the direct correlation functions.

Variational derivatives for the excess free energy of the dispersion interactions

The variational derivatives for the excess free energy related to the dispersion interactions under the high-temperature expansion – outlined in the main text – are given as follows.

Starting with the variational derivative for first-order correction, the mean-attractive energy is given by

$$\begin{aligned}\frac{\delta \mathcal{F}_{\text{att}}}{\delta \rho_i(\boldsymbol{r})} = &\sum_{j=\text{w},+,-} \left(\int \mathrm{d}\boldsymbol{r}' \left\{ \rho_j(\boldsymbol{r}') C(\lambda_1, \lambda_2) \epsilon_{ij} \left[g_{\text{hs},0}^{(i,j)}(\lambda_1)\psi(\lambda_1) - g_{\text{hs},0}^{(i,j)}(\lambda_2)\psi(\lambda_2) \right] \right\} \right) \\ &+ \frac{1}{2} \sum_{j=\text{w},+,-} \left(\int \mathrm{d}\boldsymbol{r}' \left\{ \rho_i(\boldsymbol{r})\rho_j(\boldsymbol{r}') C(\lambda_1, \lambda_2) \epsilon_{ij} \left[\frac{\delta g_{\text{hs},0}^{(i,j)}(\lambda_1)}{\delta \rho_i(\boldsymbol{r})} \psi(\lambda_1) - \frac{\delta g_{\text{hs},0}^{(i,j)}(\lambda_2)}{\delta \rho_i(\boldsymbol{r})} \psi(\lambda_2) \right] \right\} \right),\end{aligned} \tag{S11}$$

and the derivative for the second-order correction, relating to fluctuations in the local compressibility, is given by

$$\begin{aligned}\frac{\delta \mathcal{F}_{\text{fluc}}}{\delta \rho_i(\boldsymbol{r})} = &-\sum_j 2\pi \frac{\epsilon_{ij}^2}{k_\text{B}T} \frac{K_{\text{hs}}\rho_j C^2 \sigma_{ij}^3}{\lambda_2 - 3} \left(g_{\text{hs}}^{(i,j)} + \eta \frac{\partial g_{\text{hs}}^{(i,j)}}{\partial \eta} \right) \\ &-\sum_{ij} \pi \frac{\epsilon_{ij}^2}{k_\text{B}T} \frac{\rho_i \rho_j C^2 \sigma_{ij}^3}{\lambda_2 - 3} \frac{\partial K_{\text{hs}}}{\partial \rho_i} \left(g_{\text{hs}}^{(i,j)} + \eta \frac{\partial g_{\text{hs}}^{(i,j)}}{\partial \eta} \right) \\ &-\sum_{ij} \pi \frac{\epsilon_{ij}^2}{k_\text{B}T} \frac{K_{\text{hs}}\rho_i \rho_j C^2 \sigma_{ij}^3}{\lambda_2 - 3} \left(2\frac{\partial g_{\text{hs}}^{(i,j)}}{\partial \rho_i} + \eta \frac{\partial^2 g_{\text{hs}}^{(i,j)}}{\partial \rho_i \partial \eta} \right).\end{aligned} \tag{S12}$$

Results: Model calibration and demonstration

Demonstrating FMT and Wertheim's statistical association theory in capturing density profiles near hard walls

Fig. S1 demonstrates FMT's ability to reproduce density profiles of (a) hard spheres and (b) hard-sphere mixtures near hard walls. Despite the simplicity of the theory, FMT provides excellent agreement with Monte Carlo simulation results, particularly near contact, and for a wide range of probed bulk densities. The results for the bi-disperse mixture presented in S1(b) is particularly remarkable considering that only the FMT measures of the particles are required to differentiate between the two particles' profiles.

Next, Fig. S2 shows profiles in which the fluids are endowed the ability to associate

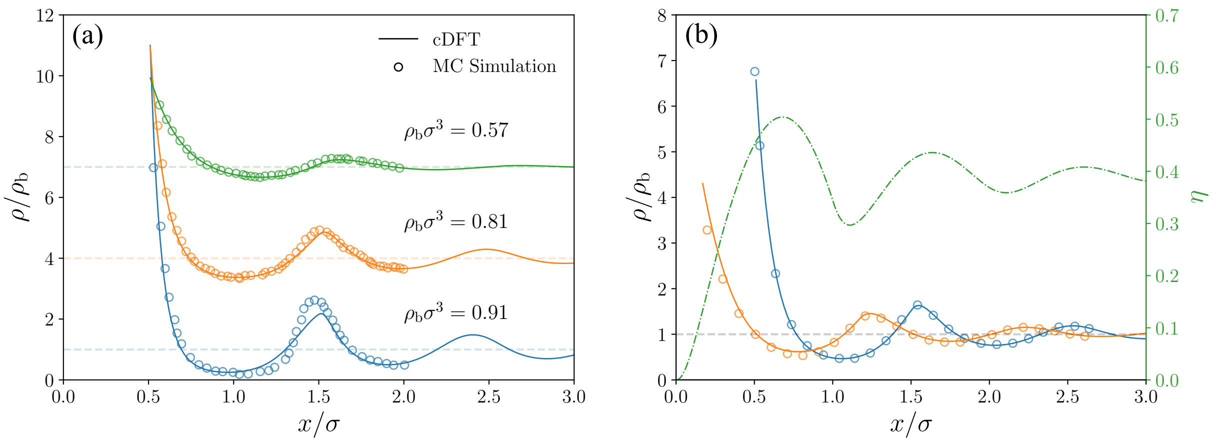

Figure S1: Normalized density profiles for hard-spheres near a hard-wall predicted using DFT (lines) and MC simulations (symbols): (a) Plots for systems of bulk densities $\rho_b\sigma^3 = 0.57, 0.81$, and 0.91; curves are vertically offset by 3 units to show the detail of the structure. MC data taken from Ref.[S9] (b) Comparison of density profiles for a bi-disperse mixture of small (s) and large (l) particles with bulk densities of $\rho_{b,s}\sigma_l^3 = 0.243$ and $\rho_{b,l}\sigma_l^3 = 0.737$, respectively, and a size ratio of $\sigma_s : \sigma_l = 1 : 3$; the green dash-dotted line plots the combined packing density $\eta = n_3$ measured along the secondary axis. MC data extracted from Ref.[S10]

with one another. Specifically, the profiles test the 4C association scheme often used to model the hydrogen bonding interactions between water molecules. These systems, which are significantly more difficult to model than the hard-sphere systems, show differences between the results of the Monte Carlo simulations and the theory implementing Yu-Wu and iSAFT models for the inhomogeneous free energy. The Yu-Wu model tends to suppress density peaks more than the iSAFT model. The Monte Carlo results are more difficult to match at high densities and strong inter-association, $\epsilon_{e_0 \cdot H}$. Nonetheless, both models are able to capture dominant trends in the profiles as the association energy is increased.

Model fitting to represent liquid-vapor coexistence and surface temperature measurements of water

Liquid-vapor (LV) coexistence at fixed T is defined by the bulk densities that share the same chemical potential, $\mu_w^L = \mu_w^V$, and bulk pressure, $P_b^L = P_b^V$. We calculate the bulk chemical potential using Eq.(4) for a single component water system in a charge-neutral

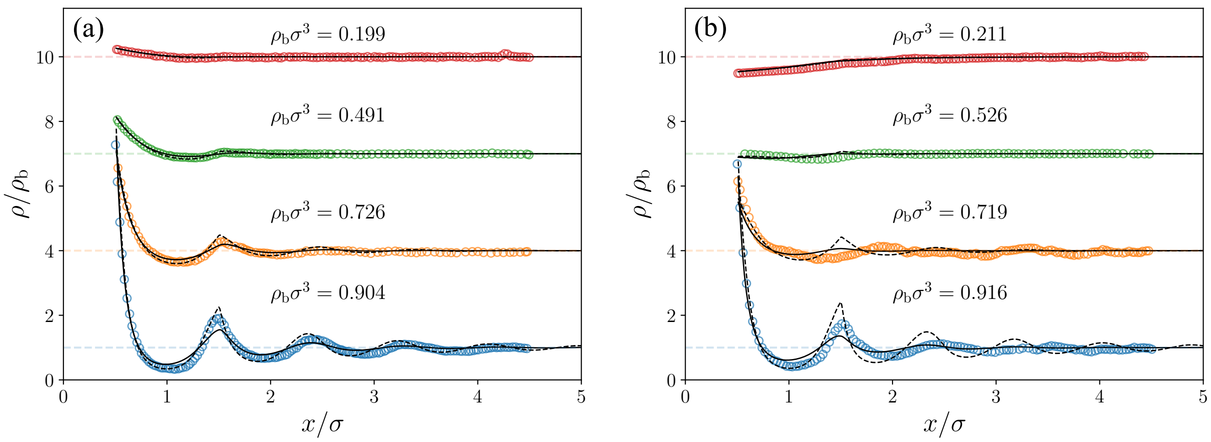

Figure S2: Density profiles for monodisperse hard-sphere associating fluid systems near a hard wall. Particles adhere to the 4C bonding scheme defined in Ref.[S11] with association energies of (a) $\beta\epsilon_{e_0 \cdot H} = 5$ and (b) $\beta\epsilon_{e_0 \cdot H} = 7$; circular markers correspond to MC simulation results,[S2,S12] solid lines correspond to the cDFT model incorporating the Yu-Wu association scheme and dashed lines correspond to the cDFT model incorporating the iSAFT association scheme. Curves are vertically offset by 3 units to show detail in the structure of the profiles.

environment, where $\mathcal{F}_{\rm es} = 0$. Under similar conditions, the bulk pressure results from the usual thermodynamic relation,

$$P_{\rm b} = -\Phi + \mu_{\rm w}\rho_{\rm w,b}, \tag{S13}$$

where Φ is the total free energy density.

Figure S3(a) shows the liquid-vapor coexistence curves for the cDFT model when adopting either the MF or HT assumptions for the dispersion interactions. Although optimization schemes may be devised to calibrate model parameters, we instead initiated the parameters to values that did well to reproduce the coexistence curves for similar cDFT models (see, *e.g.*, Ref.[S14]) and manually tuned the values to achieve our fits; the chosen parameters are listed in Table 1. Both model variants achieve excellent matches to experimental values for the LV densities over a broad range of temperatures. The coexistence curve's approach of the critical point is significantly improved by the HT expansion.

Next, the surface tension is calculated by setting the left and right boundaries to the liquid and vapor coexistence densities, respectively, and solving for the continuous transition between the two states. The equilibrium density profile thus allows the surface potential to

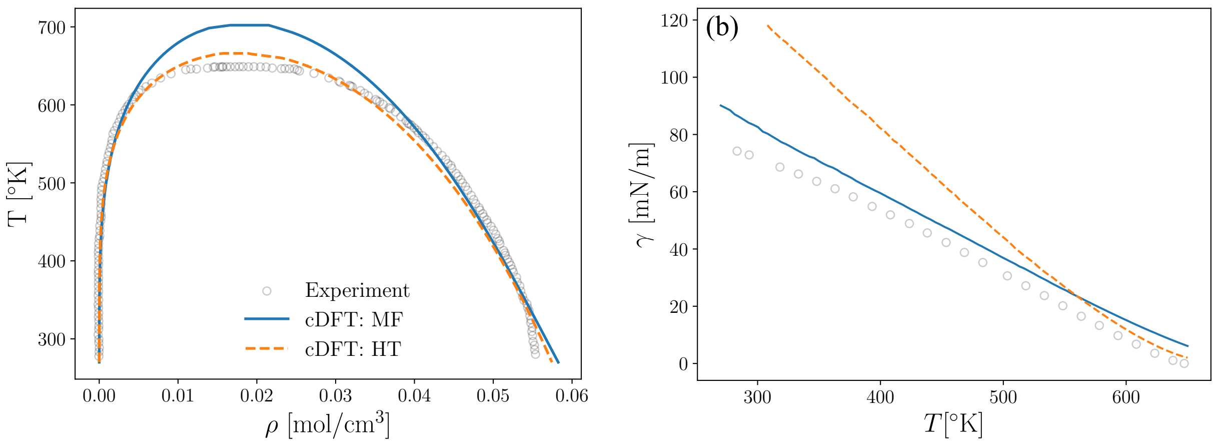

Figure S3: (a) Temperature-density plot for liquid-vapor co-existence. Curves are fitted by varying the associative and dispersive interaction parameters and equilibrating the chemical potentials and pressure in the liquid and vapor phases; optimized parameters are listed in Table 1. (b) Temperature dependent surface tension for cDFT:MF and cDFT:HT. Experimental data was collected from Ref.[S13]

be measured from the relation

$$\gamma = \frac{\Omega + P_{\mathrm{b}} V}{A}, \tag{S14}$$

where A is the interfacial surface area.

Without further adjusting the model parameters, we plot the surface tension across the LV interface as a function of T in Figure S3(b). The values of the calculated surface tension show reasonable comparison to experimental data, demonstrating similar trends and magnitudes. As expected the HT scheme performs better than the MF scheme near the critical point, though the MF scheme shows closer comparison to the experimental data over the range of probed temperatures. A better match of the HT scheme might be achieved by optimizng the parameters to match both the LV coexistence curves and surface tension.[S14]

MSA-model of electrostatic free energy near interfaces

Figure S4 demonstrates the ability of the inhomogeneous version of the MSA free energy model to incorporate out-of-plane electrostatic correlations into the ion density profiles. Excellent agreement between Monte Carlo simulations is achieved for a wide range of surfaces

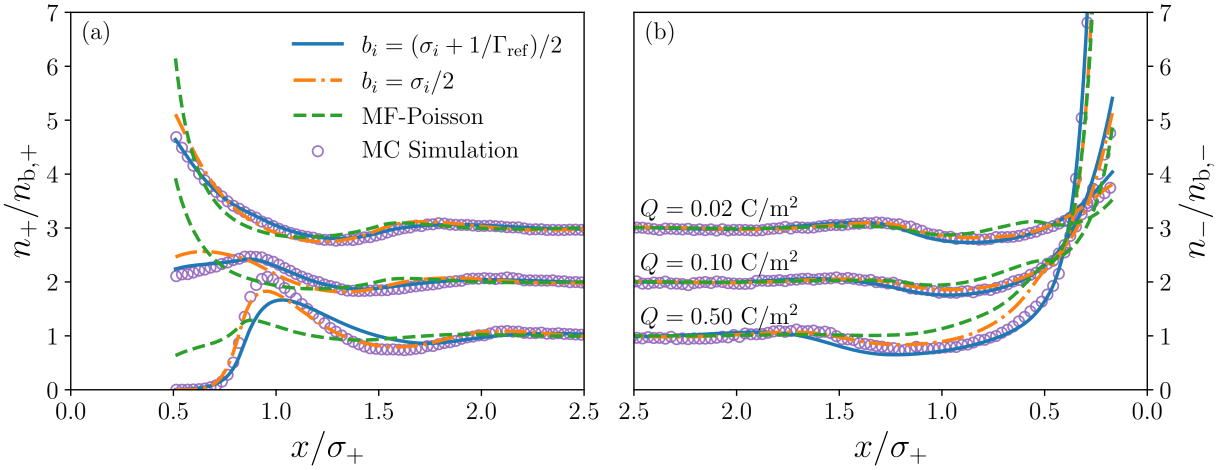

Figure S4: Comparison between different adaptations of Roth and Gillespie's charged-shell model for the MSA electrostatic energy.[S15] The double layer profile is reported for (a) the divalent cations of size $\sigma_+ = 9$ Å and (b) the monovalent anions of size $\sigma_- = 3$ Å close to positively charged hard walls; three surface charges are selected and indicated in the figure, and the bulk salt concentration is 1 M. Curves are vertically offset for clarity. Purple circular markers indicate MC simulation results[S16] and the three lines indicate Roth and Gillespie model using charged shells (continuous blue) whose capacitance radius depends on the bulk screening length of the ions or (dash-dotted orange) whose radius is set equal to the radius the radius of the ions. The last, dashed green line indicates the ion density profiles predicted by the mean-field Poisson equation discounting any MSA contribution to the electrostatic free energy.

charge values and polydispersity. T radius over which the charge of the ions is *smeared*, b_i, is chosen either as the radius of the the ions, $b_i = \sigma_i/2$, or the capacitance radius of the ions in the bulk solution, $b_i = (\sigma_i + 1/\Gamma_{\rm ref})/2$.

Figure S5 provides plots of the disjoining pressure between between impinging, charged interfaces as a function of their separation, L. The plots consider a bulk electroltye concentration of $\rho_{+,\rm b} = 0.01\,\rm M$ and plot pressures for increasing surface charge densities. The systems contain no water molecules (and are thus dilute at large surface separation) and contain no association or dispersion interactions between ions; the systems measure only the constributions of steric and Coulomb interactions.

The model demonstrates the model behavior as the electrostatic coupling between counterions and the external wall potential increases. This coupling is measured by the *electrostatic*

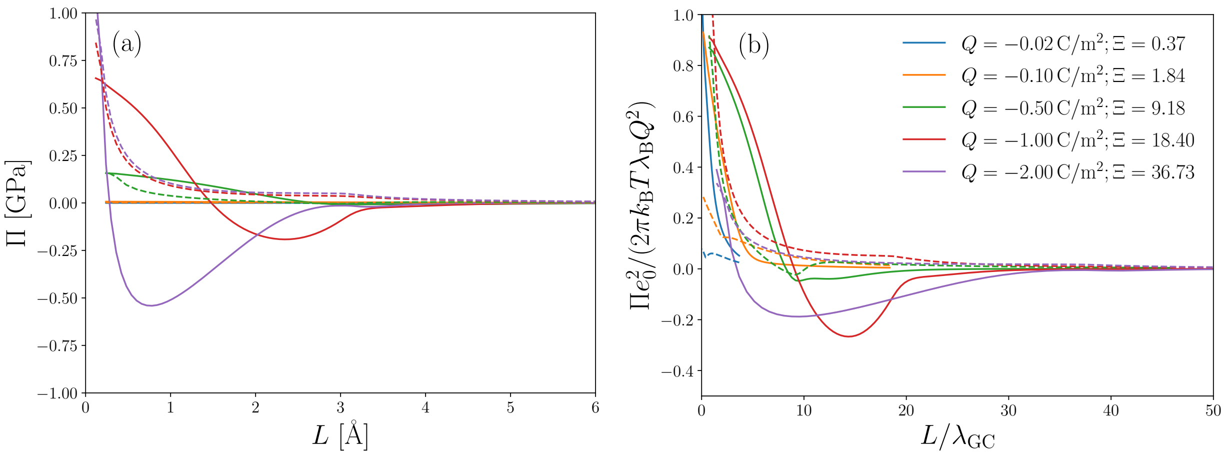

Figure S5: Disjoining pressure plotted as a function of interface separation for (a) dimensional quantities and (b) dimensionless quantities. In panel (b), the interface separation is normalized by the Gouy-Chapman length, which measures the length at which the counterion wall attraction equals the thermal energy of the ion: $\lambda_{\mathrm{GC}} = e_0(2\pi z_+ \lambda_{\mathrm{B}} Q)^{-1}$. Solid lines indicate results in which the MSA electrostatic contribution to the free energy is included and dashed lines indicate results in which the MSA contribution is neglected.

coupling parameter,

$$\Xi = \frac{z_+^2 \lambda_{\mathrm{B}}}{\lambda_{\mathrm{GC}}}, \tag{S15}$$

which compares the rescaled Bjerrum length $z_+^2\lambda_{\mathrm{B}}$ to the Gouy-Chapman length, $\lambda_{\mathrm{GC}} = e_0(2\pi z_+ \lambda_{\mathrm{B}} Q)^{-1}$. At low coupling, $\Xi \ll 1$, thermal energy dominates and the mean-field Poisson-Boltzman theory holds. At strong coupling, $\Xi \gg 1$, the ion-wall coupling dominates[S17] and additional care must be taken to account for electrostatic correlations.

Notably, the model is unable to capture the in-plane electrostatic correlation between surface bound counterions that occur at strong coupling: The out-of-plane correlations lead to significant inter-attractive pressures between surfaces, however, this pressure is significantly subdued compared to the predictions obtained using Monte Carlo simulations of point-charges and charged hard-spheres in counter-ion only systems.[S17,S18] As seen in Figure S5(a,b), the inter-attraction between like-charged surfaces disappears entirely when the MSA contribution to the electrostatic free energy is omitted entirely from the model. The inability of our model to capture in-plane correlations is due to in-plane averaging of the density profiles.

References

(S1) Gil-Villegas, A.; Galindo, A.; Whitehead, P. J.; Mills, S. J.; Jackson, G.; Burgess, A. N. Statistical associating fluid theory for chain molecules with attractive potentials of variable range. *The Journal of Chemical Physics* **1997**, *106*, 4168–4186.

(S2) Segura, C. J.; Chapman, W. G.; Shukla, K. P. Associating fluids with four bonding sites against a hard wall: density functional theory. *Molecular Physics* **1997**, *90*, 759–772.

(S3) Camacho Vergara, E. L.; Kontogeorgis, G. M.; Liang, X. A new study of associating inhomogeneous fluids with classical density functional theory. *Molecular Physics* **2020**, *118*, e1725668.

(S4) Mansoori, G.; Carnahan, N. F.; Starling, K.; Leland Jr, T. Equilibrium thermodynamic properties of the mixture of hard spheres. *The Journal of Chemical Physics* **1971**, *54*, 1523–1525.

(S5) Boublík, T. Hard-sphere equation of state. *The Journal of chemical physics* **1970**, *53*, 471–472.

(S6) Bymaster, A.; Chapman, W. G. An i SAFT density functional theory for associating polyatomic molecules. *The Journal of Physical Chemistry B* **2010**, *114*, 12298–12307.

(S7) Reed, T. M.; Gubbins, K. E. *Applied statistical mechanics*; McGraw-Hill, 1973.

(S8) Michelsen, M. L.; Hendriks, E. M. Physical properties from association models. *Fluid Phase Equilibria* **2001**, *180*, 165–174.

(S9) Snook, I. K.; Henderson, D. Monte Carlo study of a hard-sphere fluid near a hard wall. *The Journal of Chemical Physics* **1978**, *68*, 2134–2139.

(S10) Noworyta, J. P.; Henderson, D.; Sokołowski, S.; Chan, K.-Y. Hard sphere mixtures near a hard wall. *Molecular Physics* **1998**, *95*, 415–424.

(S11) Huang, S. H.; Radosz, M. Equation of state for small, large, polydisperse, and associating molecules. *Industrial & Engineering Chemistry Research* **1990**, *29*, 2284–2294.

(S12) Patrykiejew, A.; Sokolowski, S.; Henderson, D. The structure of associating fluids restricted by permeable walls: a density functional approach. *Molecular Physics* **1998**, *95*, 211–218.

(S13) Fletcher, D. A.; McMeeking, R. F.; Parkin, D. The United Kingdom chemical database service. *Journal of Chemical Information and Computer Sciences* **1996**, *36*, 746–749.

(S14) Gloor, G. J.; Jackson, G.; Blas, F.; Del Rio, E. M.; De Miguel, E. Prediction of the vapor- liquid interfacial tension of nonassociating and associating fluids with the SAFT-VR density functional theory. *The Journal of Physical Chemistry C* **2007**, *111*, 15513–15522.

(S15) Roth, R.; Gillespie, D. Shells of charge: a density functional theory for charged hard spheres. *Journal of Physics: Condensed Matter* **2016**, *28*, 244006.

(S16) Valiskó, M.; Kristóf, T.; Gillespie, D.; Boda, D. A systematic Monte Carlo simulation study of the primitive model planar electrical double layer over an extended range of concentrations, electrode charges, cation diameters and valences. *AIP Advances* **2018**, *8*, 025320.

(S17) Moreira, A. G.; Netz, R. R. Strong-coupling theory for counter-ion distributions. *Europhysics Letters* **2000**, *52*, 705.

(S18) Šamaj, L.; Trulsson, M.; Trizac, E. Strong-coupling theory of counterions with hard cores between symmetrically charged walls. *Physical Review E* **2020**, *102*, 042604.
